# Lipidated PrRP31 metabolites are long acting dual GPR10 and NPFF2 receptor agonists with potent body weight lowering effect

**DOI:** 10.1038/s41598-022-05310-y

**Published:** 2022-02-01

**Authors:** Flora Alexopoulou, Esben Matzen Bech, Søren Ljungberg Pedersen, Ditte Dencker Thorbek, Ulrike Leurs, Lise Christine Biehl Rudkjær, Keld Fosgerau, Henrik H. Hansen, Niels Vrang, Jacob Jelsing, Lisbeth Elster

**Affiliations:** Gubra, Hørsholm Kongevej 11B, 2970 Hørsholm, Denmark

**Keywords:** Metabolic disorders, Peptides

## Abstract

Prolactin-releasing peptide (PrRP) is an endogenous neuropeptide involved in appetite regulation and energy homeostasis. PrRP binds with high affinity to G-protein coupled receptor 10 (GPR10) and with lesser activity towards the neuropeptide FF receptor type 2 (NPFF2R). The present study aimed to develop long-acting PrRP31 analogues with potent anti-obesity efficacy. A comprehensive series of C18 lipidated PrRP31 analogues was characterized in vitro and analogues with various GPR10 and NPFF2R activity profiles were profiled for bioavailability and metabolic effects following subcutaneous administration in diet-induced obese (DIO) mice. PrRP31 analogues acylated with a C18 lipid chain carrying a terminal acid (C18 diacid) were potent GPR10-selective agonists and weight-neutral in DIO mice. In contrast, acylation with aliphatic C18 lipid chain (C18) resulted in dual GPR10-NPFF2R co-agonists that suppressed food intake and promoted a robust weight loss in DIO mice, which was sustained for at least one week after last dosing. Rapid in vivo degradation of C18 PrRP31 analogues gave rise to circulating lipidated PrRP metabolites maintaining dual GPR10-NPFF2R agonist profile and long-acting anti-obesity efficacy in DIO mice. Combined GPR10 and NPFF2R activation may therefore be a critical mechanism for obtaining robust anti-obesity efficacy of PrRP31 analogues.

## Introduction

Prolactin-releasing peptide (PrRP) belongs to a large family of RF-amide neuropeptides which modulate numerous functions including homeostatic regulation of appetite and energy balance^1^. The RF-amide family consists of five subfamilies of peptides, i.e. PrRP, neuropeptides FF (NPFF), RF-amide related peptide, kisspeptins and pyroglutamylated RF-amide peptides. These peptides share a common *C*-terminal Arg-Phe-amide motif that serves as pharmacophore for activation of their corresponding G-protein coupled receptors (GPCRs)^1^. In humans, PrRP circulates in two isoforms, PrRP20 and PrRP31, which are the only known endogenous ligands of the G-protein coupled receptor 10 (GPR10)^2^. These peptides are highly conserved between species^2^. PrRP and GPR10 have shown promise as potential central targets for obesity treatment, as demonstrated in several preclinical studies^3–8^. In support of an important role in energy homeostasis, GPR10 is abundantly expressed in cardinal neurocircuits in the brainstem and hypothalamus that regulate appetite function and energy expenditure, and therefore considered the principal mediator of the anorectic and weight loss promoting effects of PrRP^4,9–11^. Like most other RF-amide peptides, PrRP show cross-talk between the RF-amide family of receptors^1,12,13^. Thus, in addition to GPR10, PrRP is also a weak agonist for the neuropeptide FF receptor type 2 (NPFF2R)^1,14^. While NPFF2R might be an important contributor to the metabolic effects of PrRP^14–16^, PrRP and NPFF have also central sympathomimetic effects leading to increased arterial blood pressure and heart rate through an NPFF2R-dependent mechanism in rodents^17–20^.

Although the PrRP-GPR10 system might constitute an attractive therapeutic target in obesity treatment, the short plasma half-life of PrRP20 and PrRP31 prevents the potential use of these neuropeptides^6,8,21–23^. Attachment of fatty acids as albumin tags to bioactive peptides to prolong their duration of action is a common strategy to afford peptide-based therapies. In particular, this approach has been widely exploited for development of peptide hormone analogues suitable for diabetes and obesity treatment^24–26^. This concept has recently been applied to improve PrRP pharmacokinetics^3,27^. Accordingly, lipidation protractions up to 16 carbons have been shown to increase metabolic stability and enhancing weight-lowering properties of peripherally administered PrRP analogues in rodent^23,28,29^. Because C18 fatty acid derivatization further stabilizes against systemic clearance^24^, this prompted us to explore various C18 lipidation strategies to achieve long-acting PrRP31 analogues with potent body weight lowering efficacy.

## Results

### Structure–activity study of lipidated PrRP31 analogues

**Figure 1:**
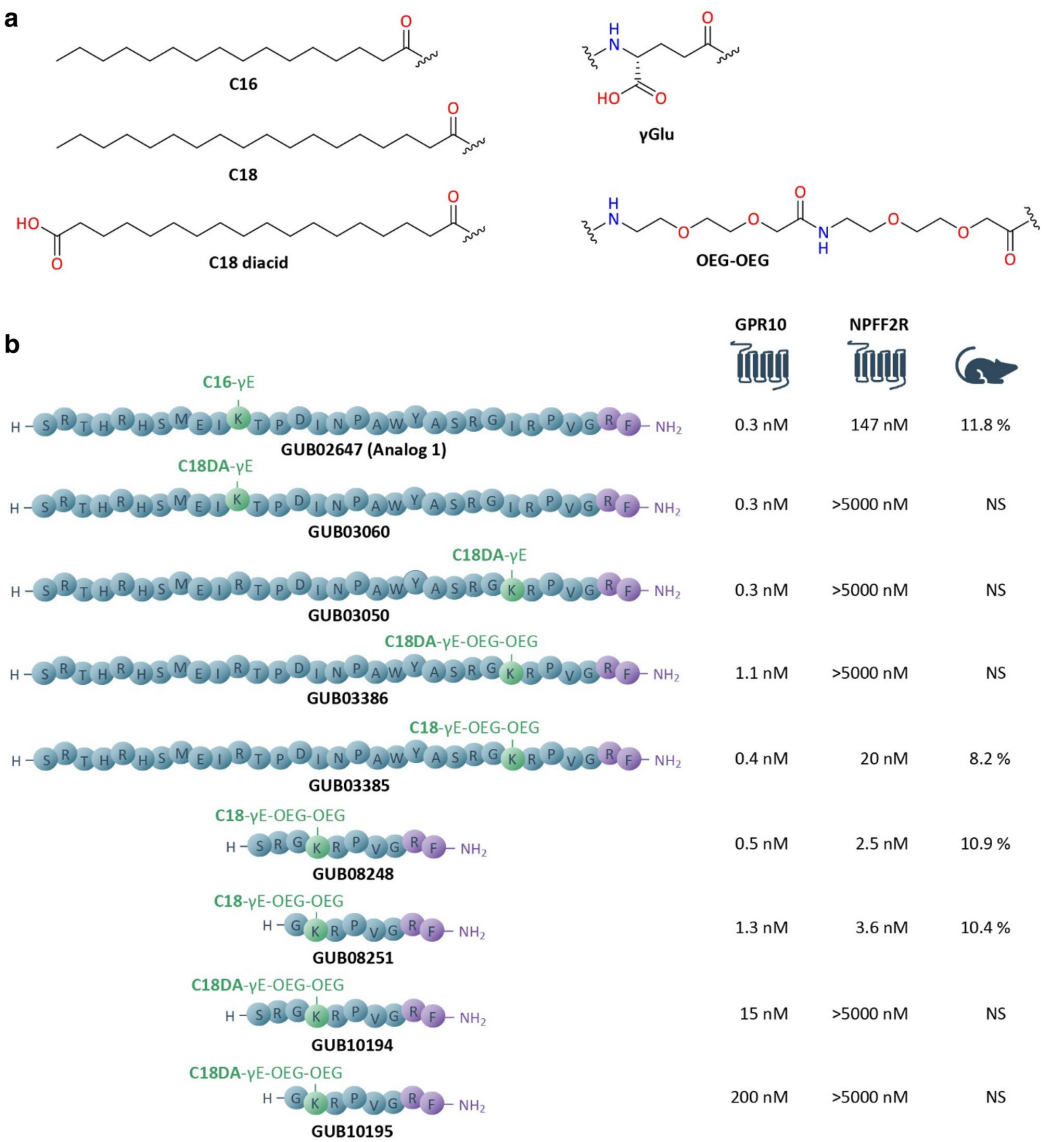
Overview of lipid chains, linkers and sequences of peptides characterized in vivo. (**a**) Protractors [C16, C18, C18-diacid (C18DA)] and linkers [γGlu (γE), OEG-OEG] applied in the current study. (**b**) Overview of sequences of PrRP31 analogues characterized in vivo. Compound EC_50_ values are indicated for GPR10 and NPFF2R activity as well as weight loss efficacy in DIO mice (% compared to baseline). NS, not significant.

A comprehensive series of lipidated PrRP31 analogues were profiled for functional GPR10 vs. NPFF2R activity, with a subset of analogues selected for further characterization in DIO mice (Fig. 1).

Whereas native PrRP20 and PrRP31 were potent GPR10-selective agonists, neuropeptide SF (NPSF) was selective for NPFF2R (Table 1). Consistent with a previous report on C16-γE-lipidated PrRP31 analogues^30^, GUB02647 (Analog 1) was similarly potent on GPR10 (EC_50_ 0.3 nM) with weak NPFF2R activity (EC_50_ 147 nM), see Table 1. Next, a comprehensive structure–activity relationship (SAR) study was performed with the aim to evaluate peptide protraction effects on GPR10 and NPFF2R activation. First, C16 was replaced with a C18 diacid (C18DA) fatty acid to yield GUB03060. Secondly, the optimal lipidation site at the PrRP31 peptide backbone was investigated by performing an extended lipidation scan followed by profiling for GPR10 and NPFF2R functional activity. As shown in Table 1, all C18DA-γE lipidated PrRP31 analogues were potent GPR10-selective full agonists (GPR10, EC_50_ 0.2–0.6 nM; NPFF2R, EC_50_ > 5 µM). Since, backbone lipidation close to the *C*-terminus of PrRP was assumed to prevent the enzymatic degradation of its *C*-terminal pharmacophore moiety, GUB03050 was selected for further optimization by introducing a γGlu-OEG-OEG spacer together with C18 (GUB03385) or C18DA (GUB03386) acylation (Fig. 1, Table 1). While GUB03385 was a dual agonist for GPR10 (full agonist, EC_50_ 0.4 nM) and NPFF2R (partial agonist, EC_50_ 20 nM), GUB03386 maintained high GPR10 selectivity and potency (EC_50_ 1.1 nM), see Table 1 and Fig. 1.

**Table 1:** Peptide sequences and GPR10/NPFF2R activity of lipidated PrRP31 analogues. Data from native PrRP isoforms (PrRP20, PrRP31) are inserted as reference.

Peptide	GPR10 EC_50_ (IP1)	NPFF2R EC_50_ (cAMP)	Amino acid sequence																															Lipid chain			
	nM	nM																																			
PrRP20	0.2	> 5000												T	P	D	I	N	P	A	W	Y	A	S	R	G	I	R	P	V	G	R	F				
PrRP31	0.2	> 5000	S	R	T	H	R	H	S	M	E	I	R	T	P	D	I	N	P	A	W	Y	A	S	R	G	I	R	P	V	G	R	F				
NPSF	> 5000	5.6																					S	Q	A	F	L	F	Q	P	Q	R	F				
GUB02647	0.3	147	S	R	T	H	R	H	S	M	E	I	K*	T	P	D	I	N	P	A	W	Y	A	S	R	G	I	R	P	V	G	R	F	C16	γE		
GUB03060	0.3	> 5000	S	R	T	H	R	H	S	M	E	I	K*	T	P	D	I	N	P	A	W	Y	A	S	R	G	I	R	P	V	G	R	F	C18DA	γE		
GUB03059	0.2	> 5000	S	R	T	H	R	H	S	M	E	I	R	T	P	D	K*	N	P	A	W	Y	A	S	R	G	I	R	P	V	G	R	F	C18DA	γE		
GUB03058	0.4	> 5000	S	R	T	H	R	H	S	M	E	I	R	T	P	D	I	K*	P	A	W	Y	A	S	R	G	I	R	P	V	G	R	F	C18DA	γE		
GUB03057	0.3	> 5000	S	R	T	H	R	H	S	M	E	I	R	T	P	D	I	N	P	K*	W	Y	A	S	R	G	I	R	P	V	G	R	F	C18DA	γE		
GUB03056	0.3	> 5000	S	R	T	H	R	H	S	M	E	I	R	T	P	D	I	N	P	A	K*	Y	A	S	R	G	I	R	P	V	G	R	F	C18DA	γE		
GUB03055	0.5	> 5000	S	R	T	H	R	H	S	M	E	I	R	T	P	D	I	N	P	A	W	K*	A	S	R	G	I	R	P	V	G	R	F	C18DA	γE		
GUB03054	0.3	> 5000	S	R	T	H	R	H	S	M	E	I	R	T	P	D	I	N	P	A	W	Y	K*	S	R	G	I	R	P	V	G	R	F	C18DA	γE		
GUB03053	0.3	> 5000	S	R	T	H	R	H	S	M	E	I	R	T	P	D	I	N	P	A	W	Y	A	K*	R	G	I	R	P	V	G	R	F	C18DA	γE		
GUB03052	0.3	> 5000	S	R	T	H	R	H	S	M	E	I	R	T	P	D	I	N	P	A	W	Y	A	S	K*	G	I	R	P	V	G	R	F	C18DA	γE		
GUB03051	0.5	> 5000	S	R	T	H	R	H	S	M	E	I	R	T	P	D	I	N	P	A	W	Y	A	S	R	K*	I	R	P	V	G	R	F	C18DA	γE		
GUB03050	0.3	> 5000	S	R	T	H	R	H	S	M	E	I	R	T	P	D	I	N	P	A	W	Y	A	S	R	G	K*	R	P	V	G	R	F	C18DA	γE		
GUB03385	0.4	20	S	R	T	H	R	H	S	M	E	I	R	T	P	D	I	N	P	A	W	Y	A	S	R	G	K*	R	P	V	G	R	F	C18	γE	OEG	OEG
GUB03386	1.1	> 5000	S	R	T	H	R	H	S	M	E	I	R	T	P	D	I	N	P	A	W	Y	A	S	R	G	K*	R	P	V	G	R	F	C18DA	γE	OEG	OEG

K*, Nε-acylated lysine; C16, hexadecenoic acid; C18DA, octadecanedioic acid; C18, octadecanoic acid; γE, γ-glutamic acid; OEG, 8-amino-3,6-dioxaoctanoic acid. All peptides were *C*-terminally amidated.

### In vivo evaluation of lipidated PrRP31 analogues

**Figure 2:**
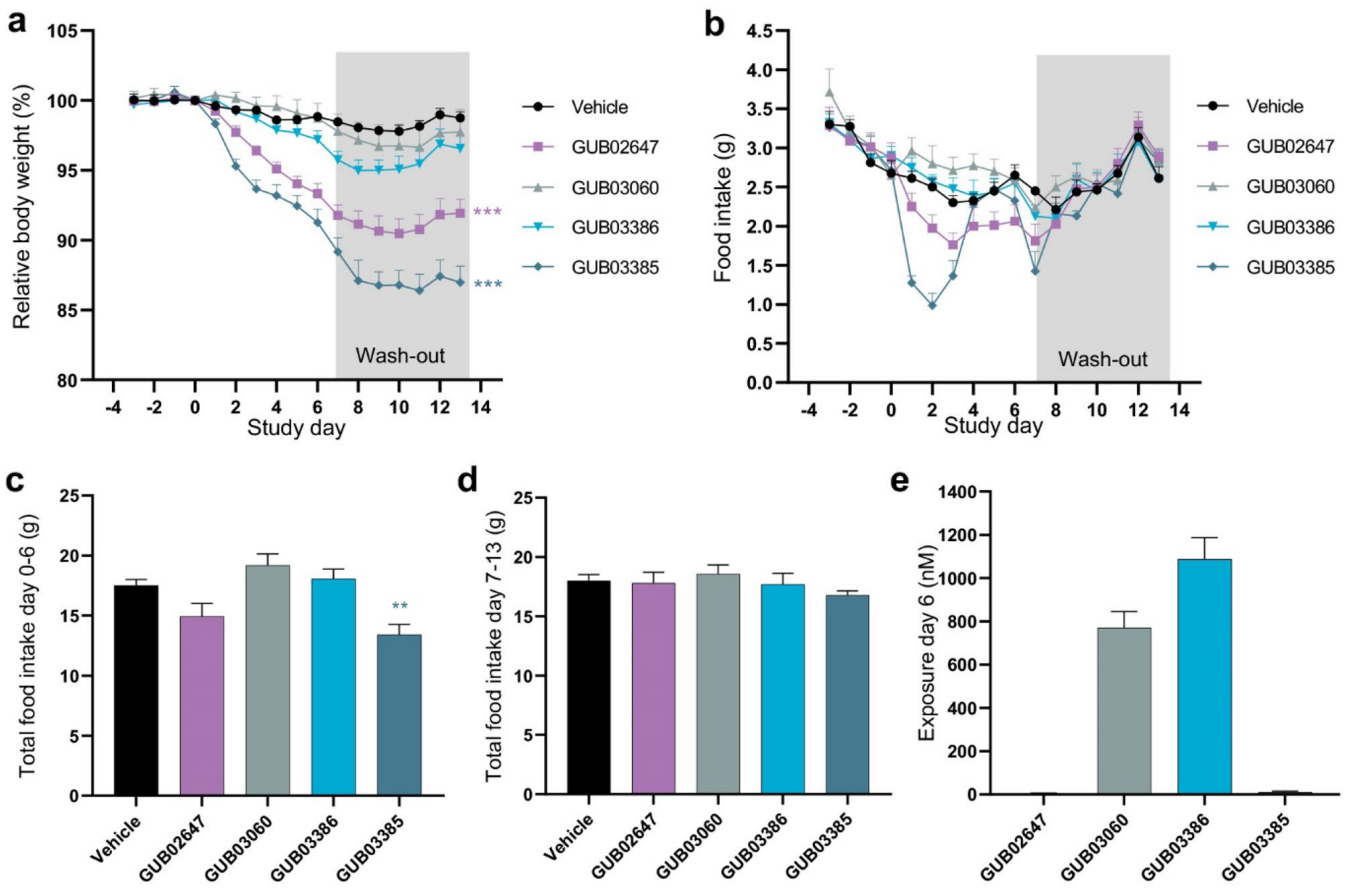
Lipidated PrRP31 analogues robustly reduce body weight and food intake in DIO mice. Effect of subchronic treatment (days 0–6) with C16/C18-lipidated PrRP31 analogues (GUB02647, GUB03385) and C18DA lipidated PrRP analogues (GUB03060, GUB03386) followed by a 7-day wash-out period (days 7–13, marked in grey). (**a**) Body weight. (**b**) 24 h food intake. (**c**) total food intake during treatment phase. (**d**) total food intake during wash-out phase. (**e**) Plasma peptide concentrations as measured 4 h after last dosing (treatment day 6). Peptides were administered (SC, BID) at a dose of 1250 nmol/kg (n = 7–8 per group). **p < 0.01, ***p < 0.001 compared to vehicle.

Metabolic effects of selected C18DA-lipidated PrRP analogues with GRP10 selectivity (GUB03050, GUB03060, GUB03386) were compared to C16/C18-lipidated PrRP analogues with dual GPR10-NPFF2R agonists profile (GUB02647, GUB03385) in DIO mice. When administered subcutaneously once daily for seven days, GUB02647 and GUB03385 induced significant body weight loss (11.8 ± 1.0%, *p* < 0.001; 8.2 ± 0.7%, *p* < 0.001), see Fig. 2a. Notably, DIO mice treated with GUB02647 and GUB03385 exhibited sustained body weight loss during the entire 7-day wash-out period (day 13; GUB02647, 8.1 ± 1.0%, *p *< 0.001; GUB03385, 13.0 ± 1.2%, *p* < 0.001). GUB02647 and GUB03385 significantly suppressed food intake during the treatment period, but not in the wash-out phase (Fig. 2b–d). No significant body weight loss and food intake inhibition was observed after treatment with the GPR10 selective C18DA-lipidated PrRP analogues GUB03386 (day 7, 4.2 ± 0.6%, *p* = 0.078; day 13, 3.4 ± 0.9%, *p* = 0.416), GUB03050 (day 7, 3.2 ± 1.1%, *p* = 0.855; day 13, 2.7 ± 2.2%, *p* = 1.000) and GUB03060 (day 7, 2.1 ± 1.2%, *p* = 0.942; day 13, 2.3 ± 1.6%, *p* = 0.906), See Fig. 2a.

### Identification of GUB03385 metabolites in vivo

**Figure 3:**
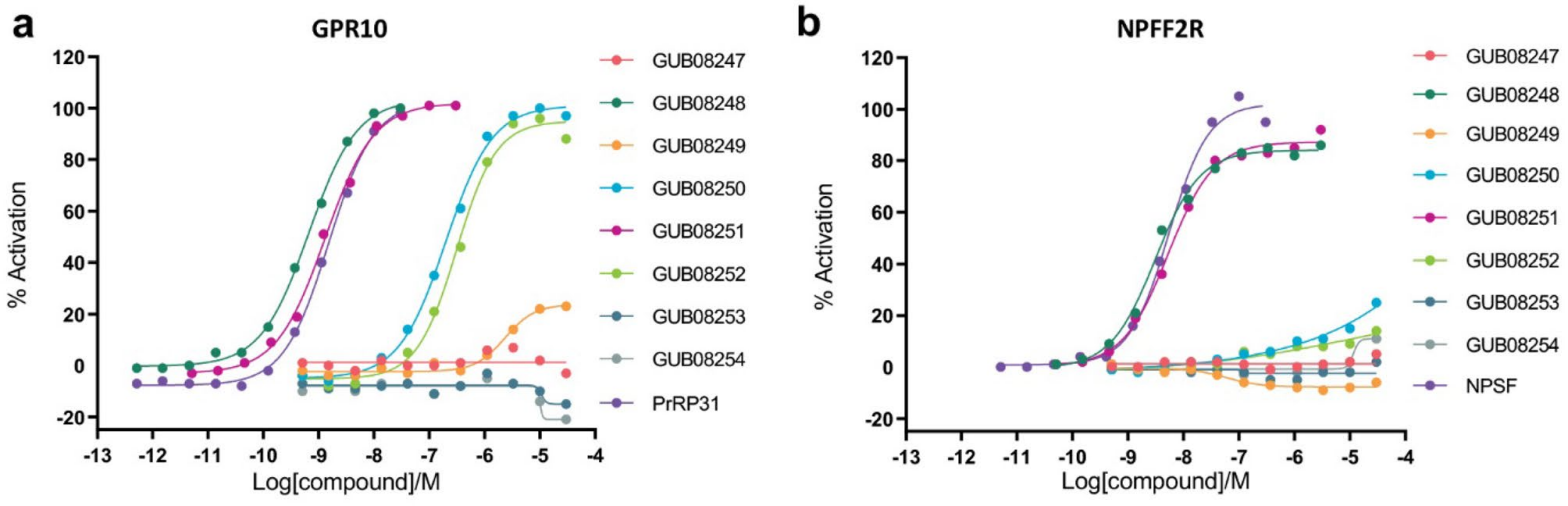
GPR10 and NPFF2R profiling of in vivo metabolites of GUB03385. (**a**) IP1 measurement in human GPR10 Chem 1 cells (HTRF technology, CisBio) after 30 min. incubation at 37 °C with metabolites and the native peptide PrRP31. (**b**) cAMP measurements in human NPFF2R CHO-K cells (HTRF technology, CisBio) at a fixed concentration of 3 µM forskolin after 30 min. incubation at room temperature with metabolites and the native peptide NPSF. Representative curves of three determinations.

GUB02647, GUB03385, GUB03060 and GUB03386 were subsequently evaluated for plasma exposure 4 h after last dosing on treatment day 6. Whereas weight-neutral C18DA-lipidated PrRP analogues (GUB03060, GUB03386) demonstrated significant plasma exposure in DIO mice, the weight-lowering non-DA C16/C18-lipidated PrRP analogues (GUB02647 and GUB03385) were not detected in plasma (Fig. 2e). A MetID study was therefore performed to determine main circulating metabolites of GUB03385. GUB03385 demonstrated rapid degradation after subcutaneous administration (< 30 min) resulting in 24 PrRP metabolites with retained C18 lipidation. Next, eight of the most abundant lipidated metabolites (highest %AUC) were synthesized for evaluation of GPR10 and NPFF2R potencies (compound series GUB08247-GUB08254, Table 2). As for the parent peptide, GUB08248 and GUB08251 demonstrated equally high potency on GPR10 (full agonists, EC_50_ 0.5 nM and 1.3 nM) and NPFF2R (partial agonists, EC_50_ 2.5 nM and 3.6 nM) (Table 2, Fig. 3). In contrast, C18DA lipidation of GUB08248 (GUB10194) and GUB08251 (GUB10195) resulted in GPR10-selective full agonists albeit with lower GPR10 potency (Table 2, Fig. 3).

**Table 2:** Peptide sequences, plasma exposure and GPR10/NPFF2R in vitro activity of in vivo metabolites of GUB03385. GUB10194 and GUB10195 are C18DA-lipidated isoforms of GU08248 and GUB08251, respectively.

Peptide	%AUC			GPR10 EC_50_ (IP1)	NPFF2R EC_50_ (cAMP)	Amino acid sequence											Lipid chain			
	30 min	60 min	120 min	nM	nM															
GUB08247	34	7	1	> 5000	> 5000	A	S	R	G	K*	R	P	V	G			C18	γE	OEG	OEG
GUB08248	11	5	n.d	0.5	2.5		S	R	G	K*	R	P	V	G	R	F	C18	γE	OEG	OEG
GUB08249	18	9	7	> 5000	> 5000		S	R	G	K*	R	P	V	G			C18	γE	OEG	OEG
GUB08250	48	23	14	200	> 5000		S	R	G	K*	R	P	V				C18	γE	OEG	OEG
GUB08251	nd	7	8	1.3	3.6				G	K*	R	P	V	G	R	F	C18	γE	OEG	OEG
GUB08252	nd	11	10	320	> 5000					K*	R	P	V	G	R		C18	γE	OEG	OEG
GUB08253	nd	11	15	> 5000	> 5000					K*	R	P	V	G			C18	γE	OEG	OEG
GUB08254	nd	5	11	> 5000	> 5000					K*	R	P	V				C18	γE	OEG	OEG
GUB10194	–	–	–	15	> 5000		S	R	G	K*	R	P	V	G	R	F	C18DA	γE	OEG	OEG
GUB10195	–	–	–	200	> 5000				G	K*	R	P	V	G	R	F	C18DA	γE	OEG	OEG

n.d., not detected; AUC, area-under-the-curve; K*, N^ε^-acylated lysine; C16, hexadecanoic acid; C18DA, octadecanedioic acid; C18, octadecanoic acid; γE, γ-glutamic acid; OEG, 8-amino-3,6-dioxaoctanoic acid.

### In vivo characterization of metabolites of lipidated PrRP31 analogues

**Figure 4:**
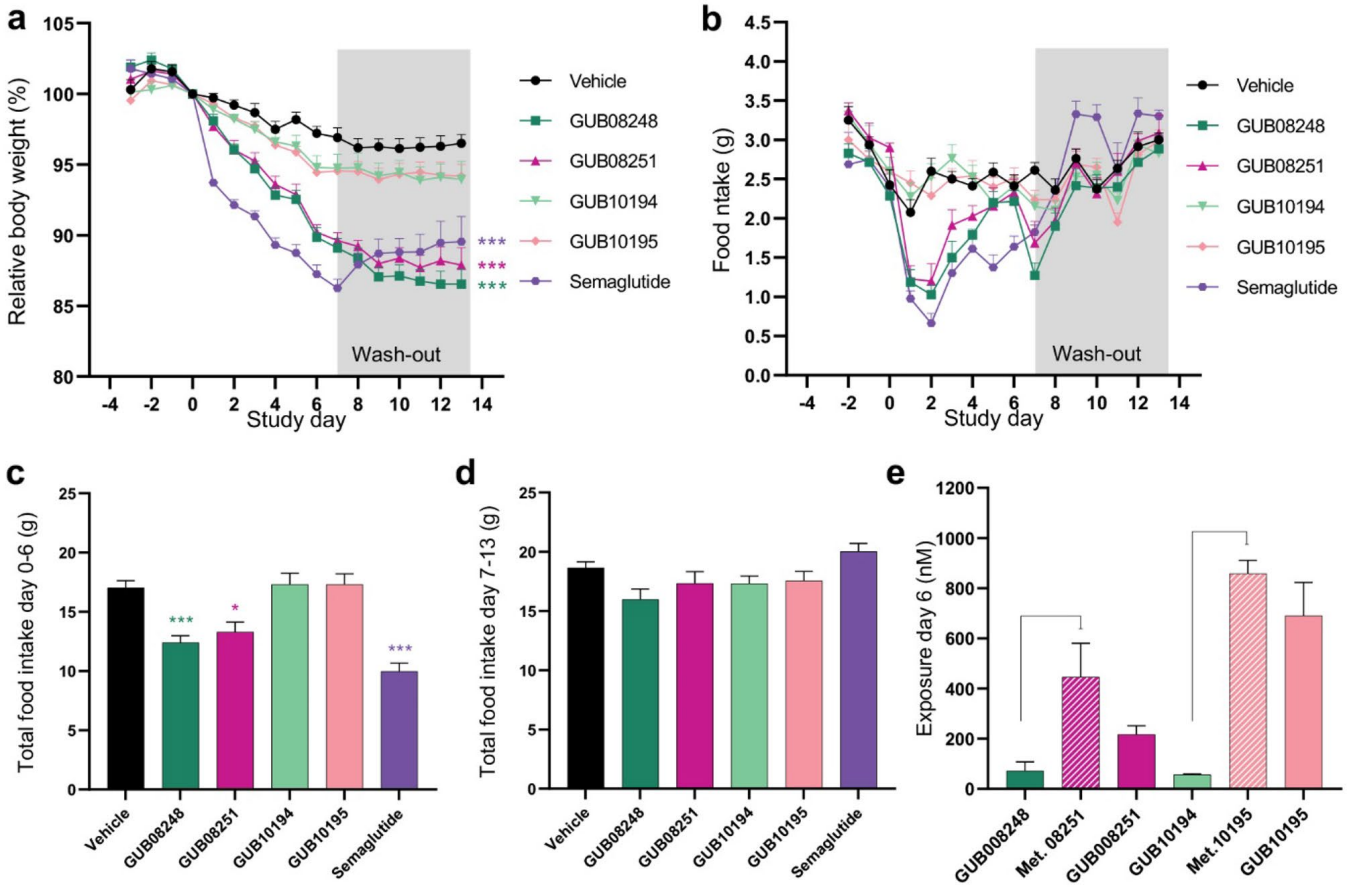
Lipidated metabolites of GUB03385 robustly reduce body weight and food intake in DIO mice. Effect of subchronic treatment (days 0–6) with GUB08248, GUB08251, GUB10194 and GUB10195 followed by 7-day wash-out period (days 7–13, marked in grey). In vivo efficacy was compared to semaglutide (10 nmol/kg, SC, QD, n = 8). (**a**) Body weight. (**b**) 24 h food intake. (**c**) Total food intake during treatment phase. (**d**) Total food intake during wash-out phase. (**e**) Plasma peptide concentrations as measured 4 h after last dosing (treatment day 6). PrRP31 analogues were administered (SC, BID) at a dose of 1,250 nmol/kg (n = 7–8 per group). *p < 0.05, ***p < 0.001 compared to vehicle.

To assess body weight lowering efficacy of GUB03385 metabolites, C18 (GUB08248, GUB08251) and C18DA (GUB10194, GUB10195) lipidated metabolites were evaluated in DIO mice (Figs. 1, 4). Compared to vehicle controls, GUB08248 induced a significant body weight loss after last dosing (day 7, 10.9 ± 0.7%, *p* < 0.001) as well as after 7 days wash-out (day 13, 13.5 ± 1.1%, *p* < 0.001). A similar efficacy was observed for GUB08251 on day 7 (10.4 ± 0.5%, *p* < 0.001) and day 13, (12.1 ± 1.2%, *p* < 0.001). As for the parent compound, both C18-lipidated GUB03385 metabolites suppressed food intake in the treatment period, but not during the wash-out phase (Figs. 2, 4). In contrast, GUB10194 and GUB010195 did not significantly influence body weight and food intake in DIO mice (Fig. 4). In comparison, semaglutide, a C18DA-lipidated GLP-1 analogue, promoted a robust body weight loss at day 7 (13.8 ± 0.6%, *p* < 0.001) which gradually wore off during the wash-out phase (day 13; 10.5 ± 1.8%, *p* < 0.001), see Fig. 4a. Semaglutide only inhibited food intake in the treatment period (Fig. 4b–d). Subsequently, GUB03385 metabolites (GUB08248 and GUB08251) and their C18DA counterparts (GUB10194 and GUB10195) were evaluated for plasma exposure 4 h after last dosing on treatment day 6. While all analogues were detected in plasma samples, higher exposure was detected for GUB08251 and GUB10195. Interestingly, GUB08248 and GUB010194 were further *N*-terminally degraded yielding GUB08251 and GUB10195, respectively (Fig. 4e).

## Discussion

The present study aimed to develop PrRP31 analogues with improved plasma stability and prolonged duration of anti-obesity action. We report that C18-lipidated PrRP31 analogues are dual GPR10-NPFF2R agonists which are rapidly degraded in vivo to PrRP31 fragments with potent and long-acting anti-obesity efficacy in DIO mice. In contrast, GPR10-selective C18DA-lipidated PrRP31 analogues were weight-neutral despite high in vivo plasma stability. Collectively, our study suggests that dual GPR10 and NPFF2R activation could be a critical determinant for anti-obesity effects of lipidated PrRP31 analogues.

We confirmed that C16-lipidation of PrRP31 through a γGlu linker at Lys11 (GUB02647/Analog 1) provides a potent GPR10 agonist with weak NPFF2R activity. Consistent with a previous report^30^, subchronic treatment with GUB02647 promoted a modest (8%) weight loss in DIO mice. To potentially further optimize in vivo efficacy of PrRP31 analogues, we applied a lipidation strategy similar to semaglutide, a long-acting C18DA-lipidated GLP-1 analogue currently approved for treatment of type 2 diabetes and in advanced clinical development for obesity^31,32^. As for the C18DA-modification of GUB02647 (GUB03060), all PrRP31 analogues carrying a C18DA protraction were GPR10-selective with in vitro potency at sub-nanomolar concentrations, irrespectively of lipid chain, acylation site, linker spacer and peptide backbone structure. The use of C18DA was therefore favourable to obtain an appropriate PrRP31 protraction without compromising GPR10 potency in vitro. In contrast, NPFF2R activity was highly affected by introducing non-DA C18-lipidation. As exemplified by GUB03385, this approach resulted in a PrRP31 analogue with dual GPR10-NPFF2R activity. Peptide lipidation-induced changes in target receptor potency have also been reported for other bioactive peptides^33–39^.

Given the highly different GPR10 and NPFF2R agonist profiles of C18DA- vs. C18-lipidated PrRP31 analogues, the two PrRP31 lipidation concepts were characterized for effects on body weight and food intake in DIO mice. Surprisingly, subcutaneous administration of GPR10-selective C18DA PrRP31 analogues (GUB03060, GUB03386) showed no significant weight reducing effect (3–4%) in DIO mice despite high in vivo plasma stability. Correspondingly, a modest weight loss (~ 5%) has recently been reported in DIO mice following peripheral administration of a half-life extended cyclic PrRP31 analogue, carrying a similar C18DA-lipidation and γGlu-OEG-OEG linker, and with selectivity towards GPR10 (67-fold GPR10:NPFF2R EC_50_ ratio)^22^. This specific lipidation protractor is identical in structure to that applied for semaglutide which promoted a robust weight loss (~ 14%) in the current study. Considering that lipidated PrRP31 analogues attached a C16 (GUB02647) or C18 (GUB03385) protraction promoted a significant weight loss (8% and 13%), this argues for the diacid lipid chain to markedly reduce in vivo efficacy of C18DA-lipidated PrRP31 despite favourable GPR10 selectivity and pharmacokinetics profile. In agreement with our data, a *N*-terminally C18-lipidated PrRP31 analogue has previously been reported to be a dual GPR10-NPFF2R agonist with appetite-suppressive effect in lean mice^23^. Also, native PrRP20 and PrRP31 have been demonstrated to exert anorectic and anti-obesity action in rodents upon central, but not peripheral, administration^4,6,8,23^. Similar effects have been reported for a series of C16-lipidated PrRP31 analogues following acute systemic administration, supporting improved CNS access of lipidated PrRP31 derivatives^23,30,40^. Considering that transport across the blood–brain barrier is generally less effective for negatively charged compounds^41^, it may be speculated that C18DA-lipidated PrRP31 analogues could have poor blood–brain barrier permeability, thereby precluding effects on body weight and food intake in DIO mice. Whether intracerebral administration of C18DA-lipidated PrRP31 analogues could afford anorectic effects must await future studies.

Notably, long-acting properties of PrRP31 analogues were maintained in both C16 and C18-lipidated PrRP31 analogues. Accordingly, both GUB02647 and GUB03385 demonstrated similar robust anti-obesity efficacy at 7 days post-dosing, although the peptides were undetectable in plasma at 4 h after last dosing. Semaglutide (10 nmol/kg) showed a different efficacy profile, as indicated by the robust weight loss gradually wearing off after treatment cessation. The rapid systemic clearance of GUB02647 and GUB03385 makes it conceivable that the anti-obesity efficacy of these PrRP analogues is not ascribed to the parent peptide, but rather determined by potent bioactive metabolite(s) with retained lipidation and high plasma stability. This prompted us to investigate the in vivo degradation products of GUB03385 in plasma following subcutaneous administration in DIO mice. Accordingly, GUB03385 showed fast degradation (< 30 min) concurrent with accumulation of several corresponding C18-lipidated metabolites. Similar to the parent peptide, a subset of truncated in vivo metabolites of GUB03385 (GUB08248 and GUB08251) with intact *C*-terminal RF-amide moiety, were dual GRP10-NPFF2R agonists. GPR10 activity of GUB03385 metabolites is in agreement with previous findings demonstrating the *C*-terminal heptapeptide segment of PrRP as the shortest sequence preserving GPR10 agonist activity^42,43^. Both GUB08248 and GUB08251 demonstrated robust and long-acting anti-obesity effect and significant plasma levels were measured 4 h after last dosing (treatment day 6). Interestingly, GUB08248 was degraded to GUB08251 suggesting that the weight-lowering properties of GUB08248 could, at least in part, be explained by metabolic actions of GUB08251. Whereas both C18DA lipidated metabolites (GUB10194 and GUB10195) were detected in plasma, C18DA lipidation rendered GUB03385 metabolites GPR10 selective and weight-neutral. Collectively, these observations argue for rapid *N*-terminal degradation of lipidated PrRP31 analogues yielding long-acting peptide fragments with robust weight-lowering efficacy linked to combined GPR10 and NPFF2R stimulation.

The food intake inhibitory effect of C18-lipidated analogues is consistent with PrRP and GPR10 expression in cardinal brainstem and hypothalamic areas regulating appetite function, including the brainstem dorsal vagal complex (nucleus tractus solitarius, area postrema, dorsal motor nucleus) and the hypothalamus (paraventricular nucleus, ventromedial nucleus)^4,7,8,44^. A role of GPR10 signalling in energy homeostasis is supported by findings that PrRP and GRP10 deficient mice display late-onset hyperphagia, adiposity and obesity^5,7,10,45,46^. It is noteworthy that both native PrRP31 and lipidated analogues promoted sustained weight loss while only having transient inhibitory effects on food intake, implying that mechanisms unrelated to appetite function were involved in the long-term weight-lowering effect. Whereas tolerance to the food intake inhibitory effects of native PrRP develops after repeated administration, PrRP has an apparent thermogenic effect following both single and repeated intracerebroventricular administration in rats^6,47,48^. The thermogenic effect of PrRP has been linked to activation of brown fat, presumably involving stimulation of brain stem and hypothalamic circuits^48,49^. Correspondingly, GPR10-deficient mice display lowered energy expenditure as determined by indirect calorimetry^5,45^. The appetite suppressing and thermogenic effects of PrRP may, at least in part, involve stimulation of central leptin signalling^8,50^. While it remains to establish similar thermogenic effects of lipidated PrRP31 analogues, stimulated expression of uncoupling protein-1, a molecular marker of brown adipose tissue (BAT) thermogenesis, has been reported after subchronic administration of a C16-lipidated PrRP31 analogue in DIO mice^30^.

The role of NPFF and NPFF2R have been much less explored in the context of obesity. NPFF2R is expressed in hypothalamic areas implicated in satiety and hunger signalling^51,52^, and central administration of NPFF has been reported to evoke dose-dependent anorectic effects in rodents^4,15,16^. As for GPR10, NPFF2R is involved in control of energy expenditure. Accordingly, NPFF2R deficient mice show exacerbated high-fat diet-induced obesity, despite lower caloric intake, due to lack of adaptive BAT thermogenesis and impaired hypothalamic neuropeptide Y signalling^53^. Interestingly, PrRP was demonstrated to exert NPFF receptor-dependent inhibition of parvocellular neuronal excitability in the hypothalamic paraventricular nucleus^20^. Collectively, NPFF2R activation could therefore potentially be implicated in the anti-obesity effects of PrRP31 analogues. However, a major impediment to define the principal molecular mechanism of PrRP31 in appetite and body weight regulation is the lack of applicable centrally acting antagonists selective for GPR10 and NPFF2R, respectively^54,55^. While GPR10 knock-out mice and neutralizing PrRP antibodies have been used to substantiate GPR10-associated anorectic effects of native PrRP31^4,7^, similar approaches have not been applied to delineate GPR10 vs. NPFF2R-mediated effects of lipidated PrRP31 analogues in vivo. It therefore remains to be elucidated whether PrRP31 analogues mediate their metabolic effects through GPR10, NPFF2R or dual GPR10-NPFF2R stimulation explains the potent anti-obesity efficacy of this compound class.

While PrRP31 analogues with dual GPR10-NPFF2R agonism might show potential as novel anti-obesity agents, preclinical evidence indicate that NPFF2R also plays a role in central cardiovascular control^55^. Major sites of NPFF2R expression are located within spinal cord and brainstem autonomic circuits regulating sympathetic outflow^51,52^. Also, both PrRP and NPFF evoke dose-dependent elevations in arterial blood pressure and heart rate in rats upon intracerebroventricular administration^17–20^. Thus, although PrRP31 analogues only exhibit partial NPFF2R agonist activity in vitro, we cannot rule out potential cardiovascular effects of these compounds. To our best knowledge, PrRP31 analogues have not been profiled for potential sympathomimetic effects, leaving it unresolved if undesirable cardiovascular responses might limit clinical utility of PrRP31 analogues.

In conclusion, C18-lipidated PrRP31 analogues are dual GPR10-NPFF2R agonists with robust and long-acting appetite-suppressive and weight-lowering efficacy in DIO mice. Protracted anti-obesity efficacy of lipidated PrRP31 analogues is most likely conferred by rapid in vivo conversion to corresponding peptide fragments with extended plasma stability and retained GPR10-NPFF2R agonist function.

## Methods

### Animals

For all in vivo studies, male C57BL/6 J mice (5 weeks old, n = 7–8) were purchased from Janvier Labs (Le Genest Saint Isle, France). Individual animals were identified by implantable subcutaneous microchips (PetID Microchip, E-vet, Haderslev, Denmark). Mice were housed in a controlled environment (12 h light/dark cycle, lights on at 3 AM, 21 ± 2 °C, humidity 50 ± 10%) and had ad libitum access to tap water and regular chow (Altromin 1324, Brogaarden, Hørsholm, Denmark) or high-fat diet (60% fat, D12492 Research diet) for up to 47 weeks prior to the initiation of the study. All animal experiments were conducted in accordance to internationally accepted principles for the care and use of laboratory animals. All experiments were approved by The Animal Experimentation Council, Danish Veterinary and Food Administration (license #2013-15-2934-00784) and the Gubra ethics committee. All experiments complied with the ARRIVE guidelines.

### Peptide synthesis

Reagents for solid-phase peptide synthesis (SPPS) were purchased from Iris Biotech GmbH (Marktredwitz, Germany), Rapp polymere GmbH (Tuebingen, Germany), AstaTech Inc. (Bristal, PA) or Sigma-Aldrich (Brøndby, Denmark). MilliQ water (Merck Millipore) was used for all experiments. Peptides were synthesized using fully automated Syro-II peptide synthesizer (MultiSynTech GmbH, Witten, Germany) by SPPS according to the 9-fluorenylmethyloxycarbonyl (Fmoc) strategy. Peptide synthesis was conducted on 0.1–0.2 nmol scale using TentaGel S RAM resin (0.24 mmol/g) as solid support. All amino acids were incorporated as standard Fmoc-protected amino acids. Fmoc-protected amino acid (4 eq.) were coupled using N,N’-diisopropylcarbodiimide (4 eq.) and ethyl (hydroxyimino)cyanoacetate (Oxyma) (4 eq.) in N,N-dimethylformamide (DMF), except Fmoc-Phe-OH which was dissolved in N-methyl-2-pyrrolidone. All couplings were performed at 75 °C for 10 min, except for His which was performed at 50 °C for 15 min, either as single or as double couplings. Fmoc deprotections were performed using piperidine in DMF, first 40% piperidine in DMF (v/v) for 3 min at 45 °C followed by 20% piperidine in DMF (v/v) for 7 min at 75 °C, except His and Asp which was deprotected at room temperature 3 min using 40% piperidine in DMF (v/v) followed by 15 min using 20% piperidine in DMF (v/v). Lipidation was performed on-resin by incorporation of Fmoc-Lys(Mtt) at the lipidation site in the amino acid sequence and N^α^-Boc protected amino acids were incorporated at the *N*-terminus to allow for full orthogonality. The Mtt group was removed using 75% HFIP in DCM for 5 × 30 min and subsequently the lipid chain was incorporated via the ε-amine of lysine using standard coupling conditions. Release of peptide from the solid support and simultaneously removal of the acid-labile side chain protecting groups was performed by incubation with a trifluoroacetic acid (TFA):triethylsilane:H_2_O (95:2.5:2.5) mixture for 35 min at 45 °C using the Razor cleavage station (CEM corp., Matthews, NC). The peptides were precipitated using cold diethyl ether. The crude peptides were purified by preparative RP-HPLC (Prep. 150-LC, Waters, Denmark) using a C8 column (5 µm, 110 Å, 21.2 × 100 mm, Phenomenex) and a solvent system containing solvent A (H_2_O + 0.1% TFA) and solvent B (acetonitrile + 0.1% TFA). B gradient elution was applied at a flow rate of 50 ml/min^-1^ and column effluent was monitored by UV absorbance at 215 nm and 254 nm. Peptide purity was determined by LC–MS. Analysis was carried out by electrospray ionization-mass spectrometry (ESI–MS) using a single quadrupole mass spectrometer (Waters, Denmark) and UPLC on an Acquity system (Waters, Denmark) system equipped with an UV detector, using a C18 column (3 μm, 110 Å, 50 mm × 4.6 mm, Waters, Denmark) and a solvent system containing solvent A (H_2_O + 0.1% formic acid) and solvent B (acetonitrile + 0.1% formic acid). A flow rate of 0.3 ml min^-1^ was applied, and column effluent was monitored by UV absorbance at 215 nm and 254 nm.

### GPR10 and NPFF2R functional activity assays

The Homogenous Time Resolved Fluorescence (HTRF) technology optimized for Gq coupled receptors has thoroughly been described in the CisBio IP1 kit manual (#62IPAPEC) and in the cAMP assay kit manual for Gi coupled receptors (#62AM9PEC). Briefly, this technic is based on a competitive immunoassay using cryptate-labelled anti-cAMP or anti-IP1 antibodies and d2-labeled cAMP or IP1. In the absence of cellular cAMP or IP1, the anti-cryptate conjugate may get into proximity to the d2 conjugate and energy (FRET) can be transferred from cryptate to d2. Chem-1 cells stably expressing the human prolactin releasing peptide receptor (GPR10, Eurofins, # HTS057C) and irradiated CHO-K1 cells stably expressing the human NPFF2R (PerkinElmer, #ES-490-CF) were applied as cells in suspension, brought to life from frozen cell stocks immediately before assay performance. 384-Well (Corning, #4513) assay formats with a total assay volume of 20 µl were applied. GPR10 cells (10.000 cells/well) were incubated with agonist peptides and the NPFF2R cells (1500 cells/well) were incubated with agonist peptides and forskolin (~ 90% activity level) for 30 min at 37 °C using IP1 kit stimulation buffer and DPBS (Sigma, #D8537) containing 0.5 mM IBMX (Sigma, # I5879), receptively. Both stimulation buffers were supplemented with 0.05% casein (Sigma, # C4765-10 ml). After addition of HTRF® detection reagents and incubation with shaking (2400 rpm) for one hour at room temperature signals at 620 and 665 nm (raw counts: ratio of 665/620) were detected at a ClarioStar plate reader (BMG Labtech, Ortenberg, Germany). Concentration–response evaluation of compounds was performed with 11 concentrations of agonist peptides (covering 3 decades) and EC_50_ values were calculated by nonlinear regression using sigmoid concentration–response with variable slope. Data were analysed using GraphPad v9.1 software (GraphPad, La Jolla, CA).

### In vivo efficacy of lipidated PrRP31 analogues in DIO mice

Compounds were freshly prepared in vehicle (PBS with 0.1% BSA, pH 4) just prior to dosing. Semaglutide was purchased from Hoersholm Pharmacy (Hoersholm, Denmark). All animals were single housed two weeks prior to treatment and throughout the remaining of the study period. Mice were randomized to treatment according to body weight (n = 7–8 per group) and dosed subcutaneously (SC, 5 ml/kg) for a total of 7 days with vehicle (BID), semaglutide (10 nmol/kg, QD) or peptide analogues (1,250 nmol/kg, BID). All compounds were administered during the light phase. Body weight and food intake (24 h) were measured daily during the treatment period and one week after treatment. In vivo data were subjected to relevant statistical analyses using GraphPad v9.1 software (GraphPad, La Jolla, CA). Statistical evaluation was carried out using Dunnett’s test one-factor linear model (endpoint body weight, food intake, cumulative food intake). A p-value less than 0.05 was considered statistically significant.

### In vivo plasma metabolite identification

Cardiac blood samples from DIO mice dosed with GUB03385 (1250 nmol/kg, SC, n = 10) were collected after 5, 10, 30, 60 and 120 min. Anaesthesia was performed using 2–4% isoflurane/O2 (Attane Vet., ScanVet Animal Health, Fredensborg, Denmark) inhalation. Blood samples were centrifuged (3000 g, 10 min) and plasma supernatants were stored at − 80 °C until MS analysis which was performed by Admescope Ltd (Oulu, Finland). Plasma precipitation was performed with twofold volume of cold acetonitrile containing 1% of formic acid. After centrifugation (16.8 g, 10 min), supernatant was pipetted to 96-well plate and analysed using a Waters Acquity UPLC coupled to a Thermo Q-Exactive Focus Orbitrap MS. Peptides were separated on a Waters CSH C18 (2.1 × 50 mm, 17.µm) column with a linear gradient from 2 to 40% acetonitrile over 3.5 min at a flowrate of 0.5 mL/min. Mass spectra were acquired in positive ionization mode in the mass range 133–2000 m/z with a resolution of 35,000; MS/MS data were acquired at a resolution of 17,500 in AIF mode with a normalized collision energy of 57. Data analysis and profiling of metabolites was performed by Admescope using Thermo XCalibur 4.1.31.9.
